# Correction: Chen et al. Deep Learning Applied to Defect Detection in Powder Spreading Process of Magnetic Material Additive Manufacturing. *Materials* 2022, *15*, 5662

**DOI:** 10.3390/ma19101911

**Published:** 2026-05-07

**Authors:** Hsin-Yu Chen, Ching-Chih Lin, Ming-Huwi Horng, Lien-Kai Chang, Jian-Han Hsu, Tsung-Wei Chang, Jhih-Chen Hung, Rong-Mao Lee, Mi-Ching Tsai

**Affiliations:** 1 Department of Mechanical Engineering, National Cheng Kung University, Tainan 701, Taiwan; 2 Department of Computer Science and Information, National Pingtung University, Pingtung 900, Taiwan; 3 Department of Intelligent Robotics, National Pingtung University, Pingtung 900, Taiwan

Following publication, concerns were raised regarding the peer-review process related to the publication of this article [1]. Adhering to our standard procedure, the Editorial Board conducted an investigation, which determined that, while the peer-review process does comply with MDPI’s Editorial process policy (https://www.mdpi.com/editorial_process), the contribution of one of the four reviewers does not comply with MDPI’s Guideline for Reviewers (https://www.mdpi.com/reviewers#_bookmark11) or the expectations of the Editorial Board. As a result, the Editorial Board has decided to remove the contribution of Reviewer 2 from the open peer-review record (https://www.mdpi.com/1996-1944/15/16/5662/review_report). Following discussion with the authors, one reference originally included at the reviewer’s suggestion has also been removed from the article [1].

The reference [22] in the original publication—Roy, A.M.; Bose, R.; Bhaduri, J. A fast accurate fine-grain object detection model based on YOLOv4 deep neural network. *Neural Comput. Appl.* **2022**, *34*, 3895–3921—has been removed. With this correction, the order of some references has been adjusted accordingly.

The authors state that the scientific conclusions are unaffected. This correction was approved by the Academic Editor. The original publication has also been updated.

## References

[1] Chen H.-Y., Lin C.-C., Horng M.-H., Chang L.-K., Hsu J.-H., Chang T.-W., Hung J.-C., Lee R.-M., Tsai M.-C. (2022). Deep Learning Applied to Defect Detection in Powder Spreading Process of Magnetic Material Additive Manufacturing. Materials.

